# Supplementary figures and images for: Smad7 Deficiency in Myeloid Cells Does Not Affect Liver Injury, Inflammation or Fibrosis after Chronic CCl4 Exposure in Mice
Source: Int J Mol Sci. 2021 Oct 27;22(21):11575. doi: 10.3390/ijms222111575 (PMC8584252; doi:10.3390/ijms222111575)

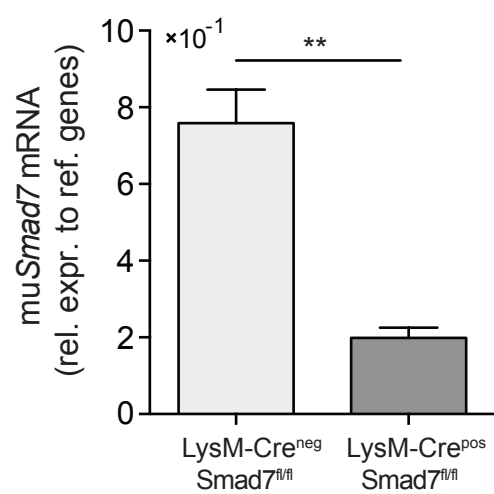

Supplement: Supplementary file 1 [file ijms-22-11575-s001.zip › ijms-1393466-supplementary.pdf]
